# Aortic dissection incidence, management, and outcomes: national population-based study

**DOI:** 10.1093/bjs/znag101

**Published:** 2026-07-31

**Authors:** Erin C Saricilar, Christian Smedberg, Rebecka Hultgren, Jacob Budtz-Lilly, Kevin Mani

**Affiliations:** Department of Surgical Sciences, Uppsala University, Uppsala, Sweden; Department of Clinical Science and Education, Södersjukhuset, Karolinska Institutet, Stockholm, Sweden; Department of Vascular Surgery, Karolinska University Hospital, Stockholm, Sweden; Vascular Surgery Group, MMK, Karolinska Institutet, Stockholm, Sweden; Department of Cardiothoracic and Vascular Surgery, Aarhus University, Aarhus, Denmark; Department of Surgical Sciences, Uppsala University, Uppsala, Sweden

## Abstract

**Background:**

Aortic dissection (AD) management requires early medical therapy and surgical intervention in selected patients with type A aortic dissection (TAAD) or complicated type B aortic dissection (TBAD). The aim of this study was to assess incidence trends of AD in Sweden, and sex-based differences in management and outcomes over 25 years.

**Methods:**

The Swedish National Patient Register and Cause of Death Register were used to identify new AD between 1999 and 2023. Incidence, management, and survival outcomes were analysed relative to the general population by matching age, sex, and year.

**Results:**

Some 15 946 individuals had AD; 12 225 were admitted to hospital alive, and, of these, 6595 (53.9%) were treated medically. Of the 5630 surgically treated individuals, 4141 (73.6%) had TAAD and 1489 (26.4%) presented with TBAD. Incidence increased significantly to 9.32 per 100 000, with an annual percentage change (APC) of 1.4% (95% c.i. 1.2% to 1.6%). Incidence was lower for women (6.25 per 100 000) than men (10.52 per 100 000), but the APC was three-fold higher in women (APC 2.3% (95% c.i. 1.9% to 2.6%)) compared with men (APC 0.8% (95% c.i. 0.5% to 1.0%)). Women presented at an older age than men (mean of 71.9 *versus* 67.5 years, *P* < 0.001), were less likely to receive surgical treatment than men (women represented 37.8% of all dissection patients, but only 33.6% of surgically treated TAAD patients and 29.3% of surgically treated TBAD patients, *P* < 0.001), and had worse 10-year relative survival than men (medical management: women 70.9% and men 84.1%, *P* = 0.0041; TAAD surgical management: women 86.6% and men 94.9%, *P* < 0.0001; and TBAD surgical management: women 70.9% and men 87.5% *P* = 0.027).

**Conclusion:**

The incidence of AD is increasing in Sweden, with persisting sex disparities in management and outcomes. These findings underscore the need for targeted interventions to improve the diagnosis, management, and prognostication of AD, especially in women.

## Teaser

This study assesses trends in incidence, management, and outcomes of aortic dissections in Sweden over a 25-year interval (1999–2023). Cross-matching of national registries was performed to identify 15 946 individuals with new-onset AD, including those medically and surgically managed, as well as pre-hospital deaths. There was an increasing incidence of dissections in Sweden during this interval (annual percentage change (APC) of 1.4%), with a three-fold increase in APC for women compared with men. Whilst early operative outcomes improved over time, women were consistently less likely to receive surgical treatment and had a lower rate of early survival. All patients with dissection had inferior relative survival up to 10 years compared with a sex-, age-, and year-matched general population. The negative effect of dissection on relative long-term survival was also more prominent in women compared with men.

## Introduction

Aortic dissection (AD) is a life-threatening vascular emergency that involves the delamination of the aortic wall layers, resulting in the formation of a false lumen through which blood can flow. It is classified according to the Stanford system or the Society for Vascular Surgery (SVS)/Society of Thoracic Surgeons (STS) reporting standards as type A aortic dissection (TAAD) or type B aortic dissection (TBAD) based on the proximal extent of the pathology relative to the distal border of the left subclavian artery and innominate artery respectively^1^. Whereas untreated TAADs have a high mortality rate and require urgent surgical intervention, TBADs can be managed medically or surgically (open or endovascular) based on their presentation and complications.

Significant gaps remain in the understanding of AD epidemiology. With a sharply decreasing incidence of aortic aneurysm disease in some countries, questions remain regarding the development of dissection pathology^2,3^. Whilst patients with aortic aneurysm and dissection pathology share several clinical characteristics, significant differences in disease pathophysiology exist that could result in different epidemiological development for these two aortic pathologies. The effect of medical and surgical development on survival after dissection, overall and relative to the general population, is not well known^4,5^. Sex-based and regional differences have been described within the AD cohort, with female patients having delayed presentation, receiving different treatments, having different outcomes, and having poorer long-term survival^6–8^.

The aim of this study was to comprehensively assess the trends in incidence of AD in Sweden over a 25-year interval, and to evaluate the management and survival of patients with AD based on sex and region.

## Methods

### Study design

Within Sweden, all residents are assigned a unique 12-digit personal registration number that includes the date of birth and a 4-digit personal identification number (PIN) as a suffix. This registration number is used to identify patients across all regions as part of the Swedish Board of Health and Welfare. An individual patient’s hospitalization and outpatient visit data are recorded in the National Patient Register (NPR). This includes ICD-9/ICD-10 codes for diagnosis and Nordic Medico-Statistical Committee (NOMESCO) Classification of Surgical Procedure (NCSP) codes for procedures. Associated dates, such as admission, diagnosis, and procedure dates, are also included.

Additionally, a Cause of Death Register (CDR) records all deaths of any person who dies in Sweden regardless of region and location of death (inpatient or outpatient). Providing a cause of death using ICD-10 codes is mandatory and this is recorded in the CDR.

To ensure quality of study design and analysis, the STROBE statement was used as a guide^9^. Ethical approval was obtained from the Regional Board of Ethical Approval of Uppsala, DNR 2017/027.

### Data collection

As this was a population-based retrospective cohort study, the NPR was utilized to identify all patients who had been diagnosed with an acute AD (ICD-10 code I71.0 or ICD-9 code 441). The time interval for data extraction was from 1 January 1994 to 31 December 2023. All included patients were deidentified. To ensure that the study only included new diagnoses of AD, a 5-year washout interval was applied from 1 January 1994 to 31 December 1998, leaving only patients from 1999 to 2023 who did not have a diagnosis of AD at any time prior. This provided a study cohort of new-onset AD for 25 years. The washout interval assumed that any patient who had a diagnosis of AD before 1994 would have at least once been registered with a code for AD in the 5-year washout interval for surveillance, intervention, follow-up, or another incidental finding as part of a different pathology. Accordingly, any patient with a new diagnosis of AD between 1999 and 2023 was deemed to have a new AD.

Demographic data were extracted, including the date of birth, sex, and co-morbidities at diagnosis. The co-morbidities were defined based on broad ICD-10 code groups related to cardiac disease, pulmonary disease, cerebrovascular disease, diabetes mellitus, chronic kidney disease, cancer, dementia, liver disease, hypertension, and peripheral arterial disease.

All procedures that were relevant to open and endovascular dissection repair were included and determined by NCSP codes. The type of AD was determined by the operative code. For patients who were medically managed, classification of the AD was unclear, as the type of dissection cannot be identified without imaging or procedural codes. Previous studies suggest that some 8.6% of acute type A dissections can be managed medically, with a 48 h mortality rate of 23.7%^10^. Similarly, a study performed in Stockholm County showed that 21% of medically managed type A dissection patients remained alive at 1 week^1^. Therefore, it was deemed inappropriate to assume that medically managed patients were exclusively TBAD patients.

The STS/SVS reporting standards for AD were used for anatomical and chronicity definitions^11^. A TAAD patient was identified as any patient with an AD commencing in zone 0 (based on surgical repair codes), whereas all surgically treated patients with aortic repair starting in zone 1 or beyond were classified as TBAD patients (including both open and endovascular surgical management). This enabled differentiation of patients who only underwent medical management as the standard of care for uncomplicated dissections and patients who were not suitable for medical management only, without bias of the operative technique preferences of different centres impacting analysis. Patients who failed to respond to medical management in the TBAD cohort were assumed to be those who underwent surgical management in the chronic interval, as they would have the uncomplicated dissections that developed an indication for surgical treatment while being managed medically.

Cross-linkage with the CDR was used to verify the date of death. Whether a person died before diagnosis in the hospital was also registered using the CDR.

Python version 3.9.6 (Python Software Foundation, Wilmington, DE, USA) was used to manipulate the NPR database to a useable format. Coding and execution of the code was performed using Visual Studio Code (Microsoft Corporation, Redmond, WA, USA) and version control was managed using Git (Software Freedom Conservancy, Brooklyn, NY, USA).

### Statistical analysis

All data analysis was performed using SPSS^®^ (IBM, Armonk, NY, USA; version 30.0) and figures were produced using Excel (Microsoft Corporation, Redmond, WA, USA) or R (R Foundation for Statistical Computing, Vienna, Austria). All data was assumed parametric for analysis due to the size of the cohort based on central limit theorem. *P* < 0.050 was deemed statistically significant before corrections were applied. Statistical significance between categorical variables was assessed using a Pearson’s chi-squared test, whereas continuous variables for more than two groups were compared using ANOVA.

National and annual incidence was calculated based off the total adult population (aged ≥18 years) of Sweden in the relevant year and reported as persons per 100 000. Population mortality probability per 1000 was extracted based on age, year, and sex. All national data were extracted using Statistikdatabasen available from Statistics Sweden (scb.se). A Poisson log-linear time-trend regression analysis was performed to estimate the annual percentage change (APC), with statistical significance determined using a Wald test, given with a 95% confidence interval. When analysing a change in outcome between two time intervals, the entire cohort was divided into year of dissection 1999–2008 and 2009–2023, as there was the most even distribution of patients who had completed a total of 10 years of follow-up.

Kaplan–Meier survival curves were used to assess survival based on sex and stratified time intervals with statistical significance assessed using a log rank test. Patients who were registered as having AD deaths without admission were removed from all survival analysis. To control for variables, demographic data (age, sex, and region of domicile) and co-morbidities (cardiac, pulmonary, cerebrovascular, chronic kidney, liver, and peripheral arterial disease, as well as diabetes mellitus, cancer, dementia, and hypertension) were included as covariates in Cox regression models and the results are presented as HRs with 95% confidence intervals. Sensitivity analysis was performed on the Cox regression models where patients aged ≥80 years were excluded to determine the true effect of sex on survival outcomes. Univariate regression was performed on all demographic and co-morbidity variables specifically for medical management, type A dissections treated surgically, and type B dissections treated surgically (open and endovascular). Bonferroni correction was performed due to the large number of independent comparators within the regression model. Standard Kaplan–Meier survival curves and landmark analysis of long-term survival up to 10 years (crude and relative to the age-, sex-, and year-matched general population) were used for patients who survived the initial 90 days after dissection. The landmark analysis was utilized to assess the effect of dissection on overall survival when excluding the acute dissection-related mortality. Sensitivity analysis was not performed on relative survival, as the impact of age was included in the analysis by matching sex, age, and year of AD.

## Results

### Patient characteristics

**Figure 1:**
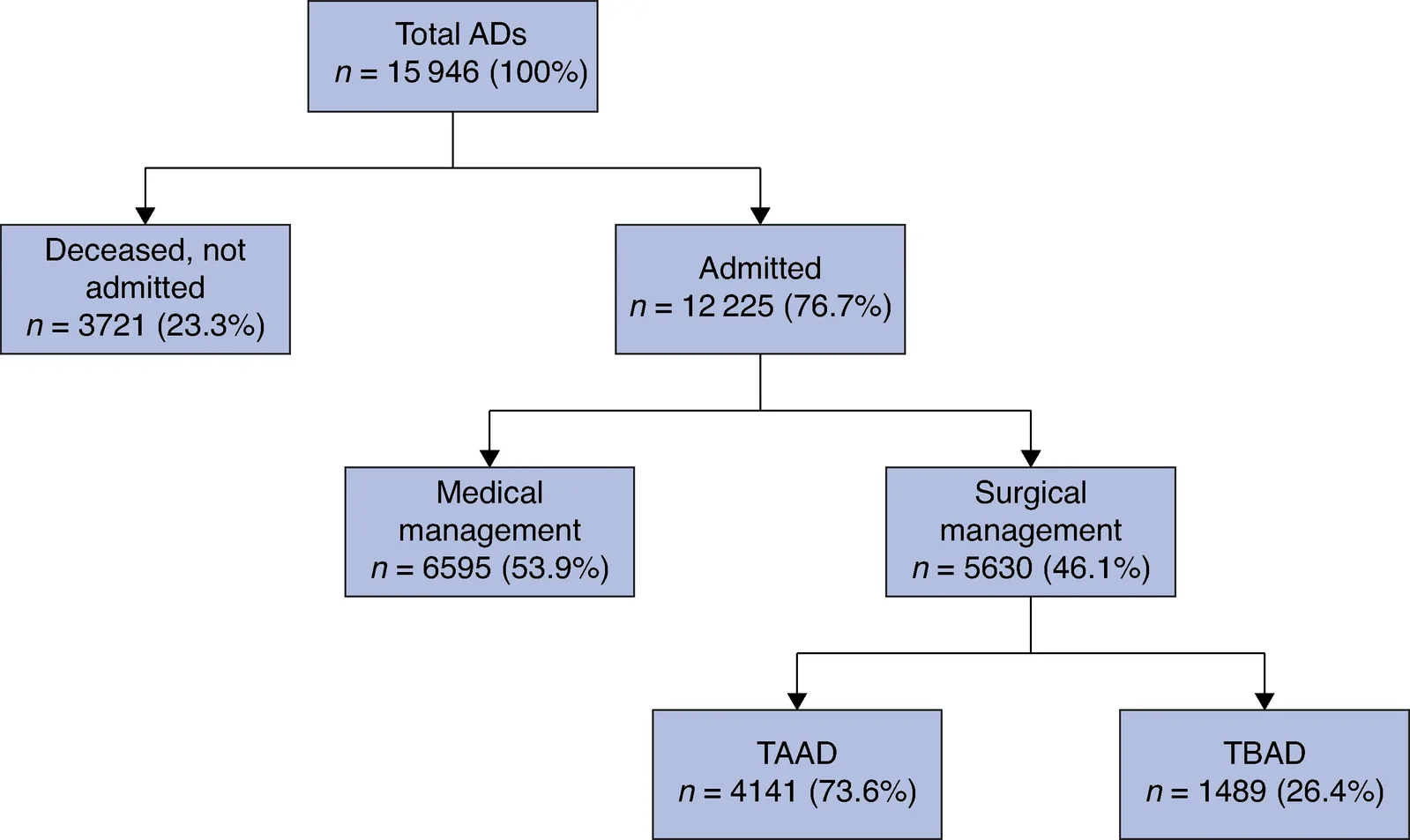
Flow chart summarizing total ADs from 1999 to 2023 The separation of cohorts reflects the methods used to delineate groups of patients based on ICD-10 codes and NOMESCO procedure coding. ADs, aortic dissections; TAAD, type A aortic dissection; TBAD, type B aortic dissection; NOMESCO, Nordic Medico-Statistical Committee.

From 1999 to 2023, a total of 15 946 ADs were registered; of the 15 946 individuals, 3721 (23.3%) died due to AD without admission to a hospital. Of the 12 225 individuals who were admitted, 6595 (53.9%) were treated medically. Of the 5630 surgically treated individuals, 4141 (73.6%) had TAAD and 1489 (26.4%) presented with TBAD (*Fig. 1*).

The mean(s.d.) age was 69.2(13.2) years. The mean(s.d.) age of surgically treated patients (TAAD 62.3(11.7) years and TBAD 64.1(12.3) years) was lower than that of those who died without admission (71.0(12.7) years) and those medically treated (72.2(12.8) years) (*P* < 0.001) (*Table 1*), with similar results when sex was separated (*Table S1* and *Table S2*). Those aged ≥80 years represented 31.5% of the medically managed cohort, 3.9% of the surgically managed TAAD patients, and 7.3% of the surgically managed TBAD patients (*Table 1*).

**Table 1:** Means and frequencies of patient factors (age, sex, region, and co-morbidities) for medical management, TAAD surgical management, TBAD surgical management, and total ADs

	Medical management	TAAD surgical management	TBAD surgical management	Total ADs	*P*
**Age (years), mean(s.d.)**	72.15(12.77)	62.3(11.70)	64.08(12.27)	69.16(13.15)	<0.001
<50	425 (5.5)	530 (14.4)	94 (10.9)	1275 (8.0)	<0.001
50–59	772 (10.0)	851 (23.1)	196 (22.7)	2269 (14.2)	
60–69	1524 (19.8)	1175 (31.9)	251 (29.1)	3787 (23.7)	
70–79	2548 (33.2)	978 (26.6)	259 (30.0)	4995 (31.3)	
80–89	2083 (27.1)	145 (3.9)	60 (7.0)	3125 (19.6)	
>90	331 (4.4)	0 (0.0)	3 (0.3)	495 (3.1)	
**Sex**					
Male	4712 (61.3)	2442 (66.4)	610 (70.7)	9911 (62.2)	<0.001
Female	2971 (38.7)	1237 (33.6)	253 (29.3)	6035 (37.8)	
**Region**					
Urban	2185 (28.5)	1151 (31.3)	195 (22.6)	4623 (29.1)	<0.001
Metropolitan	3109 (40.6)	1487 (40.5)	385 (44.7)	6497 (40.8)	
Rural	2366 (30.9)	1034 (28.2)	282 (32.7)	4790 (30.1)	
**Co-morbidities**					
Cardiac disease	4071 (53.0)	1811 (49.2)	250 (29.0)	6132 (50.2)	<0.001
Pulmonary disease	1294 (16.8)	362 (9.8)	116 (13.4)	1772 (14.5)	<0.001
Cerebrovascular disease	2040 (26.6)	848 (23.0)	172 (19.9)	3060 (25.0)	<0.001
Diabetes mellitus	770 (10.0)	170 (4.6)	43 (5.0)	983 (8.0)	<0.001
Chronic kidney disease	980 (12.8)	451 (12.3)	93 (10.8)	1524 (12.5)	0.888
Cancer	1666 (21.7)	458 (12.4)	117 (13.6)	2241 (18.3)	<0.001
Dementia	437 (5.7)	171 (4.6)	21 (2.4)	629 (5.1)	<0.001
Liver disease	383 (5.0)	159 (4.3)	40 (4.6)	582 (4.8)	0.422
Hypertension	4586 (59.7)	1861 (50.6)	509 (59.0)	6956 (56.9)	<0.001
Peripheral arterial disease	1009 (13.1)	234 (6.4)	89 (10.3)	1332 (10.9)	<0.001

Values are *n* (%) unless otherwise indicated. Statistical significance was assessed using ANOVA for continuous variables and a chi-squared test for categorical variables. TAAD, type A aortic dissection; TBAD, type B aortic dissection; ADs, aortic dissections.

Patients from urban, metropolitan, and rural regions represented 29.1%, 40.8%, and 30.1% of all dissection patients respectively. Urban patients were more likely to be surgically treated for TAAD compared with metropolitan and rural patients, whereas metropolitan and rural patients were more likely to be surgically treated for TBAD compared with urban patients. These represented significant differences based on the relative total of all dissections (*P* < 0.001) (*Table 1*), which was again similar when sex was separated (*Table S1* and *Table S2*).

The mean(s.d.) age for men and women was 67.2(13.1) and 71.2(13.1) years respectively (*P* < 0.001). Women represented 37.8% of all dissection patients, but only 33.6% of surgically treated TAAD patients and 29.3% of surgically treated TBAD patients (*P* < 0.001) (*Table 1*).

### Incidence trends

**Figure 2:**
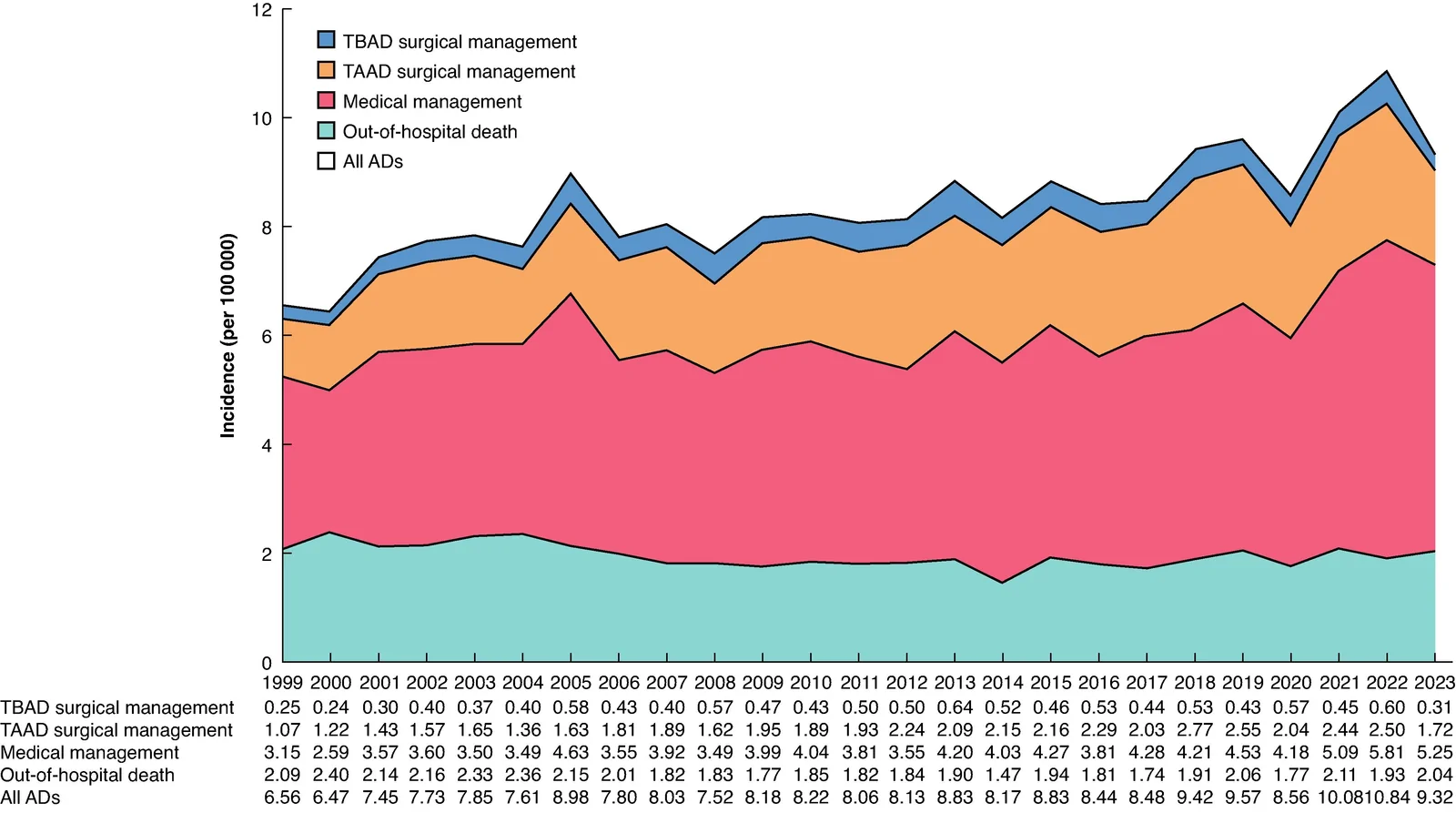
Stacked incidence of ADs per 100 000 persons per year, for cohorts of death without admission, medical management, TAAD surgical management, and TBAD surgical management A summary table of incidences is included underneath the graph. ADs, aortic dissections; TAAD, type A aortic dissection; TBAD, type B aortic dissection.

From 1999 to 2023, the total incidence of AD went from 6.56 to 9.32 people per 100 000—a mean incidence of 8.36 people per 100 000 with a significant APC of 1.4% (95% c.i. 1.2% to 1.6%) (*P* < 0.001). The mean incidence of prehospital death was 1.97 people per 100 000 with a negative APC of 0.7% (95% c.i. 1.1% to 0.2%) (*P* < 0.001). The mean incidence of medical management was 4.02 people per 100 000 with an APC of 1.9% (95% c.i. 1.6% to 2.2%) (*P* < 0.001). The mean incidence of surgical management for TAAD was 1.92 people per 100 000 with an APC of 1.9% (95% c.i. 1.6% to 2.2%) (*P* < 0.001) and the mean incidence of surgical management for TBAD was 0.45 people per 100 000 with an APC of 1.5% (95% c.i. 0.6% to 2.4%) (*P* < 0.001) (*Fig. 2*). The mean incidence of TBAD patients who failed to respond to medical therapy and underwent surgical management in the chronic interval was 0.14 people per 100 000 with a negative APC of 2.66% (95% c.i. 1.59% to 3.73%) (*P* < 0.001), representing, on average, 32.7% of all TBAD patients treated surgically.

The mean incidence of all dissections in men was 10.52 per 100 000 with a significant positive APC of 0.8% (95% c.i. 0.5% to 1.0%) (*P* < 0.001). For women, the mean incidence was 6.25 per 100 000 with a higher APC of 2.3% (95% c.i. 1.9% to 2.6%) (*P* < 0.001) (*Fig. S1*). There was less change in incidence for diagnosis of any dissection for those aged <65 years (mean incidence of 3.60 per 100 000 with an APC of 1.0% (95% c.i. 0.6% to 1.4%) (*P* < 0.001)) and for those aged ≥65 years (mean incidence of 23.67 per 100 000 with an APC of 0.8% (95% c.i. 0.6% to 1.1%) (*P* < 0.001)) (*Fig. S2*).

### Survival analyses

**Figure 3:**
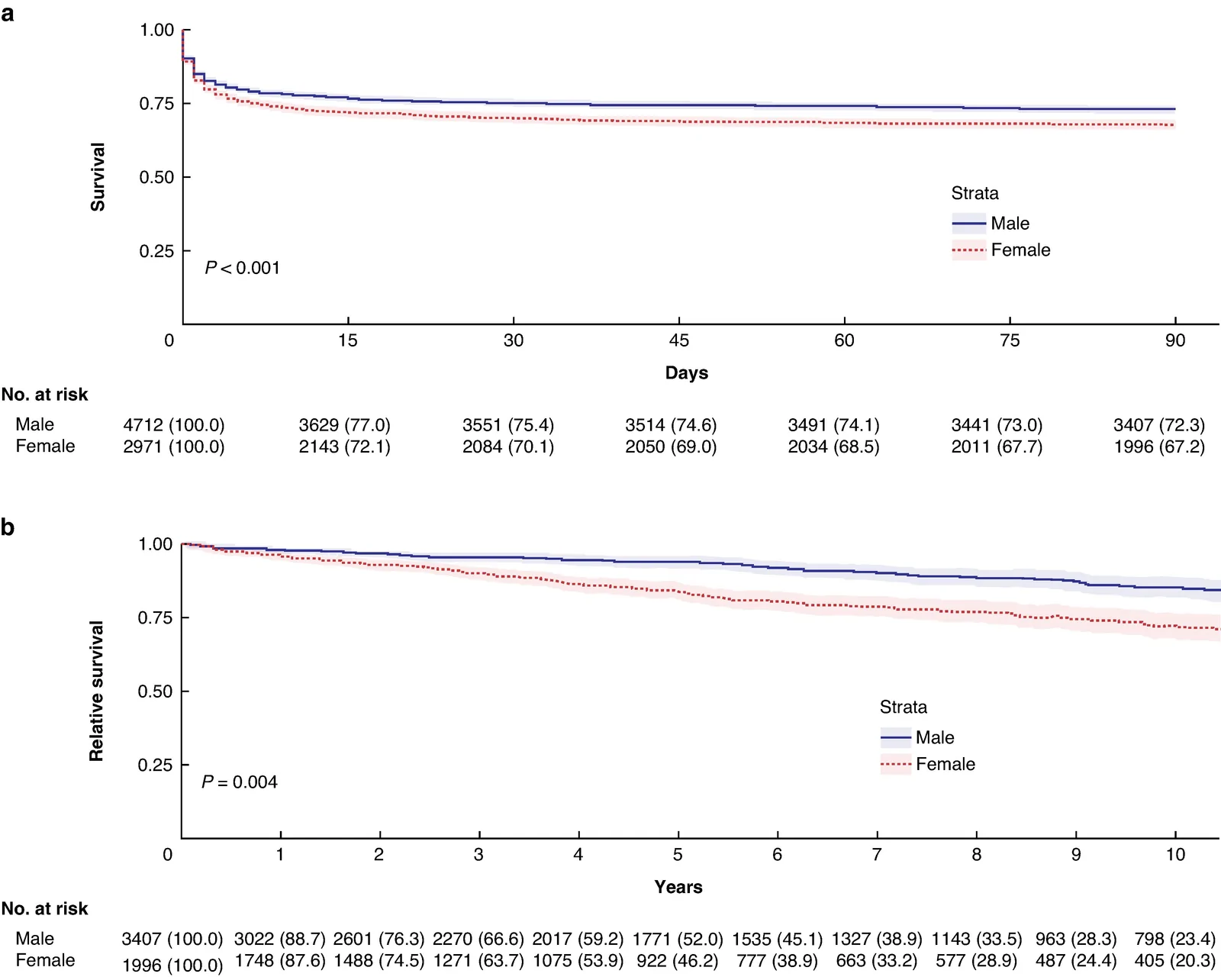
Survival of medically managed AD patients **a** Kaplan–Meier plot of 90-day survival, stratified by sex, with statistical significance assessed using a log rank test. **b** Ten-year relative survival, matched by year of dissection and age then stratified by sex, with statistical significance assessed using a log rank test. Values in parentheses are percentages. AD, aortic dissection.

Within the medical management cohort, there was a significant difference in 90-day survival between men and women (72% and 67% respectively, *P* < 0.0001). Women also had poorer 10-year relative survival (matched by age and year of dissection) compared with men (*P* = 0.004). See *Fig. 3*, *Table 2*, and *Table 3*. A standard Kaplan–Meier survival analysis (that did not adjust for age-, dissection year-, or sex-based differences in survival) showed similar crude survival for men and women up to 10 years after dissection (*Fig. S3*). When controlling for age, region of domicile, and co-morbidities using Cox regression, female sex trended as a predictor of 90-day mortality in medically managed patients (*P* = 0.005), but was not statistically significant. In a sensitivity analysis excluding patients aged ≥80 years, sex again was not a significant factor affecting 90-day survival. The difference between sexes was not significant for 10-year survival outcomes (*Table 4*).

**Table 2:** Crude survival estimates (Kaplan–Meier) at 90 days, 1 year (90-day mortalities excluded), 5 years (90-day mortalities excluded), and 10 years (90-day mortalities excluded) after dissection, stratified by medical management, TAAD surgical management, and TBAD surgical management, as well as by sex

	Ninety days	One year	Five years	Ten years
**Medical management**				
Male	0.729 (0.717,0.742)	0.942 (0.934,0.950)	0.795 (0.782,0.809)	0.722 (0.707,0.737)
Female	0.678 (0.661,0.694)	0.935 (0.924,0.946)	0.761 (0.742,0.780)	0.721 (0.702,0.741)
**TAAD surgical management**				
Male	0.842 (0.828,0.857)	0.985 (0.980,0.990)	0.924 (0.912,0.935)	0.872 (0.858,0.887)
Female	0.844 (0.824,0.864)	0.978 (0.969,0.987)	0.897 (0.878,0.915)	0.836 (0.814,0.859)
**TBAD surgical management**				
Male	0.915 (0.893,0.937)	0.978 (0.966,0.991)	0.875 (0.847,0.902)	0.799 (0.766,0.833)
Female	0.866 (0.824,0.908)	0.945 (0.915,0.975)	0.858 (0.812,0.905)	0.767 (0.711,0.823)

Values in parentheses are 95% confidence intervals. TAAD, type A aortic dissection; TBAD, type B aortic dissection.

**Table 3:** Summary of relative survival rate at 1 year, 5 years, and 10 years (90-day mortalities excluded) after dissection, stratified by medical management, TAAD surgical management. and TBAD surgical management, as well as by sex

	One year	Five years	Ten years
**Medical management**			
Male	0.986 (0.982,0.990)	0.939 (0.927,0.951)	0.841 (0.818,0.863)
Female	0.969 (0.963,0.975)	0.835 (0.817,0.853)	0.709 (0.676,0.742)
**TAAD surgical management**			
Male	1.000 (0.997,1.003)	0.996 (0.988,1.004)	0.949 (0.933,0.965)
Female	0.992 (0.987,0.997)	0.941 (0.927,0.955)	0.866 (0.839,0.893)
**TBAD surgical management**			
Male	0.999 (0.992,1.006)	0.963 (0.943,0.983)	0.875 (0.837,0.913)
Female	0.961 (0.944,0.978)	0.884 (0.846,0.922)	0.709 (0.631,0.787)

Values in parentheses are 95% confidence intervals. TAAD, type A aortic dissection; TBAD, type B aortic dissection.

**Table 4:** Summary of all patients factors and their impact on survival (90-day survival and 10-year survival when excluding 90-day mortalities) for medical management, TAAD surgical management, and TBAD surgical management

	HR (95% c.i.); *P*		
	Medical management	TAAD surgical management	TBAD surgical management
**Ninety-day survival**			
Age	1.03 (1.02,1.03); <0.001	1.03 (1.03,1.07); <0.001	1.01 (0.99,1.03); 0.287
Sex	1.13 (1.04,1.23); 0.005	0.85 (0.72,1.02); 0.079	1.55 (0.99,2.41); 0.056
Region	1.05 (0.99,1.11); 0.090	1.09 (0.98,1.21); 0.130	1.15 (0.85,1.54); 0.365
Cardiac disease	1.21 (1.10,1.32); <0.001	0.97 (0.81,1.15); 0.693	1.22 (0.750,2.00); 0.418
Pulmonary disease	1.00 (0.59,1.11); 0.947	1.11 (0.86,1.43); 0.441	1.42 (0.82,2.47); 0.215
Cerebrovascular disease	1.17 (1.06,1.28); 0.001	1.45 (1.21,1.73); <0.001	1.38 (0.83,2.31); 0.220
Diabetes mellitus	0.86 (0.74,1.00); 0.050	1.09 (0.76,1.56); 0.638	1.73 (0.78,3.86); 0.179
Chronic kidney disease	1.11 (0.98,1.25); 0.103	1.30 (1.04,1.63); 0.021	1.60 (0.89,2.88); 0.118
Cancer	1.01 (0.92,1.12); 0.789	0.85 (0.67,1.09); 0.205	0.85 (0.44,1.63); 0.621
Dementia	1.29 (1.11,1.50); 0.001	0.56 (0.35,0.89); 0.015	0.67 (0.16,2.84); 0.590
Liver disease	1.10 (0.91,1.31); 0.322	1.41 (1.01,1.99); 0.045	1.97 (0.96,4.07); 0.066
Hypertension	0.69 (0.63,0.75); <0.001	0.94 (0.79,1.12); 0.510	0.58 (0.37,0.92); 0.021
Peripheral arterial disease	0.94 (0.83,1.07); 0.333	1.15 (0.86,1.56); 0.351	1.32 (0.71,2.44); 0.379
**Ten-year survival**			
Age	1.04 (1.04,1.05); <0.001	1.03 (1.02,1.04); <0.001	1.04 (1.02,1.05); <0.001
Sex	0.59 (0.80,0.99); 0.035	1.12 (0.92,1.37); 0.270	1.04 (0.74,1.46); 0.821
Region	0.97 (0.91,1.04); 0.379	1.08 (0.96,1.22); 0.215	1.02 (0.83,1.25); 0.868
Cardiac disease	1.02 (0.92,1.14); 0.698	0.98 (0.80,1.19); 0.810	1.09 (0.77,1.56); 0.623
Pulmonary disease	1.16 (1.02,1.32); 0.023	1.31 (0.98,1.75); 0.067	1.37 (0.91,2.07); 0.129
Cerebrovascular disease	0.90 (0.80,1.02); 0.091	0.83 (0.65,1.06); 0.137	1.17 (0.80,1.71); 0.409
Diabetes mellitus	0.95 (0.80,1.13); 0.576	1.44 (0.96,2.17); 0.079	0.83 (0.39,1.80); 0.641
Chronic kidney disease	0.82 (0.69,0.97); 0.017	0.81 (0.58,1.11); 0.186	0.95 (0.57,1.57); 0.834
Cancer	0.81 (0.71,0.91); <0.001	0.73 (0.54,1.00); 0.047	0.66 (0.41,1.07); 0.093
Dementia	1.26 (1.02,1.54); 0.030	0.82 (0.51,1.32); 0.411	1.02 (0.40,2.55); 0.973
Liver disease	1.11 (0.89,1.39); 0.364	1.15 (0.73,1.80); 0.556	1.71 (0.92,3.19); 0.091
Hypertension	0.80 (0.72,0.89); <0.001	0.90 (0.74,1.10); 0.320	0.90 (0.64,1.27); 0.560
Peripheral arterial disease	1.17 (1.01,1.34); 0.033	1.29 (0.90,1.85); 1.289	1.18 (0.73,1.90); 0.501

*P* < 0.004 indicates significance with Bonferroni correction from pooled Cox regression. TAAD, type A aortic dissection; TBAD, type B aortic dissection.

**Figure 4:**
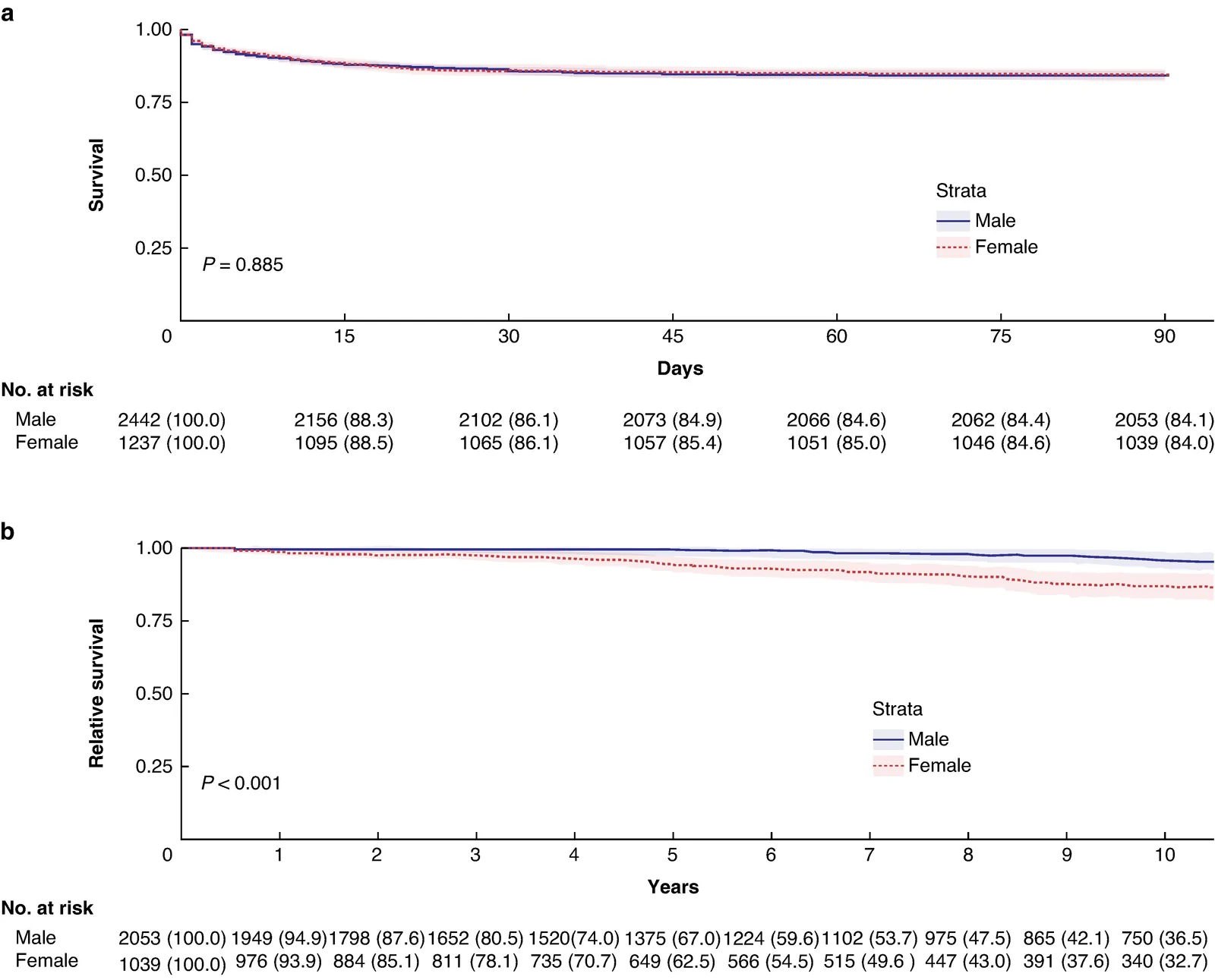
Survival of surgically managed TAAD patients **a** Kaplan–Meier plot of 90-day survival, stratified by sex, with statistical significance assessed using a log rank test. **b** Ten-year relative survival, matched by year of dissection and age then stratified by sex, with statistical significance assessed using a log rank test. Values in parentheses are percentages. TAAD, type A aortic dissection.

For surgically managed TAAD, there was no significant difference between male and female survival at 90 days. The survival for men and women remained the same in a sensitivity analysis excluding patients aged ≥80 years. Relative survival at 10 years was significantly poorer for women (*P* < 0.001). See *Fig. 4*, *Table 2*, and *Table 3*. A standard Kaplan–Meier survival curve did not demonstrate any difference in crude 10-year survival between men and women; however, landmark analysis showed that women had poorer crude survival than men (*P* = 0.001) (*Fig. S4*). When a Cox regression was performed to control for age, region of domicile, and co-morbidities, sex was not a predictor for outcomes in surgically managed TAAD (*Table 4*).

**Figure 5:**
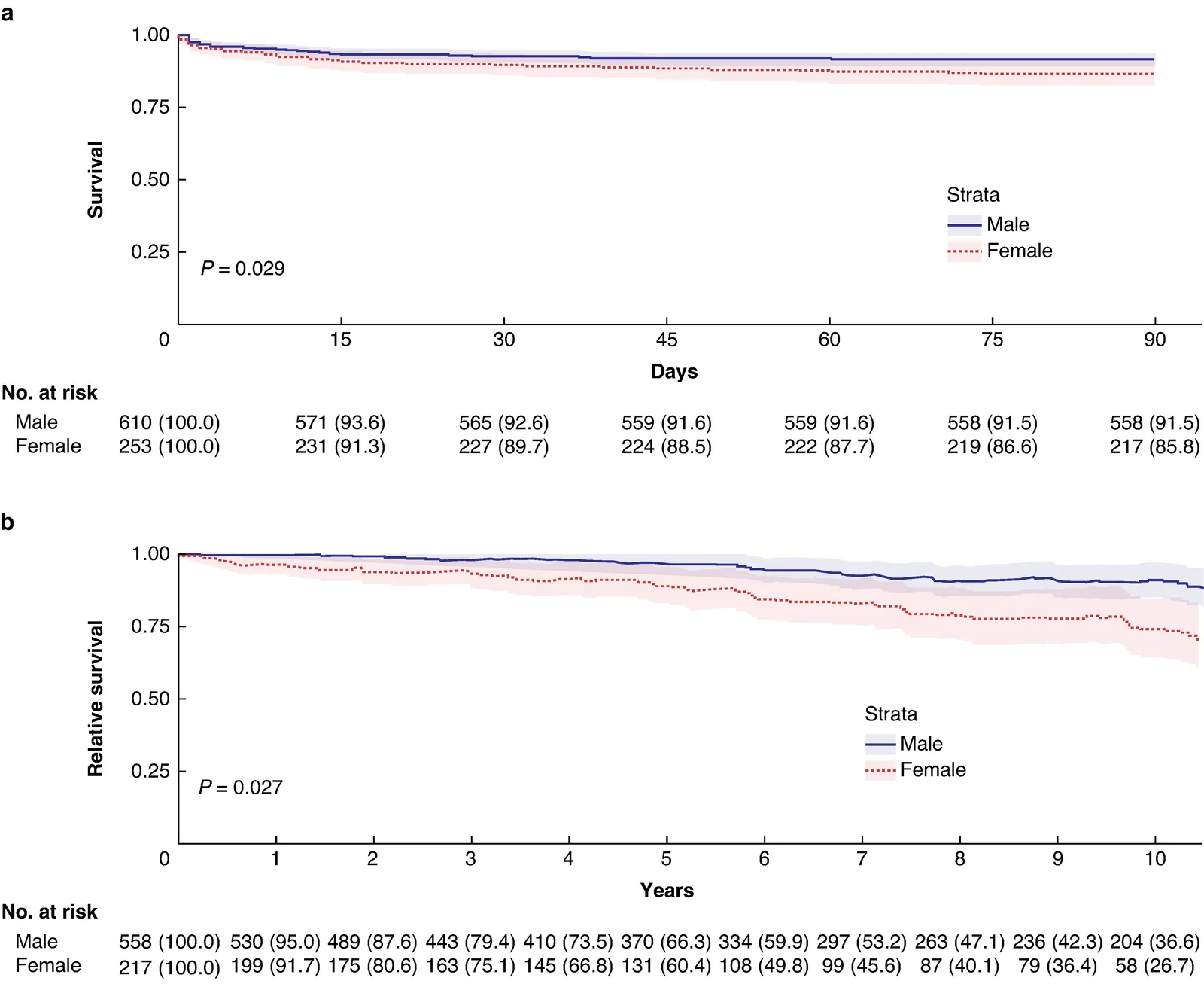
Survival of surgically managed TBAD patients **a** Kaplan–Meier plot of 90-day survival, stratified by sex, with statistical significance assessed using a log rank test. **b** Ten-year relative survival, matched by year of dissection and age then stratified by sex, with statistical significance assessed using a log rank test. Values in parentheses are percentages. TBAD, type B aortic dissection.

For surgically managed TBAD, there was a significant difference between male and female survival at 90 days (*P* = 0.029), which became insignificant when performing a sensitivity analysis excluding patients aged ≥80 years. Women did have significantly poorer relative survival at 10 years (*P* = 0.027). See *Fig. 5*, *Table 2*, and *Table 3*. A standard Kaplan–Meier survival curve did not demonstrate any difference in crude 10-year survival between men and women (*P* = 0.061), which was confirmed in the landmark analysis (*Fig. S5*). When a Cox regression was performed to control for age, region of domicile, and co-morbidities, sex was not a predictor for outcomes in surgically managed TBAD (*Table 4*).

A significant improvement in survival was observed in those diagnosed with AD in 2009 and onwards compared with the earlier cohort, for both men and women at 90 days, as well as long-term (*Fig. S6* and *Fig. S7*).

## Discussion

This 25-year nationwide population-based analysis of AD epidemiology provides new insights, showing a steadily increasing sex-stratified incidence of AD in Sweden. Women had a higher increase in the APC of AD compared with men, which might represent improved healthcare delivery to women overall. Still, women had significantly poorer relative 10-year survival, indicating that AD has a more profound impact on female patients’ expected survival than that of male patients. At the same time, women with AD are less likely to receive surgical treatment. The effect of AD on overall long-term survival is underlined by the relative survival assessment in this cohort. Even after surgical intervention, patients with AD have inferior survival compared with age-, sex-, and year-matched Swedish inhabitants, and the negative survival effect is most prominent in women. This negative effect on long-term relative survival persists in the most recent cohorts despite improvements in early outcome.

The increasing incidence of ADs is in contrast to the decreasing incidence of abdominal aortic aneurysms in Sweden over the past decade, verified in national screening studies and attributed to decreases in smoking practices^2,3,12^. This finding underlines the pathophysiological differences between dissection and aneurysmal disease of the aorta, with dissections being less dependent on smoking patterns than aneurysms^13^. The increase in incidence occurred in patients presenting to hospital with dissection, whilst the incidence of dissections identified as the cause of death without admission to hospital declined in the same interval. The increase in overall ADs is most likely due to a detection bias with the increased use of CT diagnostics during early undifferentiated workup, as well as overall increased age and expected survival in the Swedish patient population during the study interval. The use of CT imaging in Sweden increased 130% over the interval 2005 to 2018^14^. Improvements in hypertension and cardiovascular health management in the community may explain the decrease in out-of-hospital death related to AD. In the present data set, it was not possible to identify connective tissue disorders, aortopathies, and other genetic disorders that may have an impact on incidence rates, but it is unlikely that such factors would change over time. Ethnicity of patients is another factor not available in the Swedish NPR. However, previous analysis indicates that the vast majority of aortic patients in Sweden are born in Sweden (88.3%), or Europe (10.6%), with only 1.1% being non-European^15^.

Interestingly, the rate of patients managed medically *versus* surgically did not change over the years. The lack of granularity in registry data does not allow for a detailed analysis of treatment patterns for TBAD, where controversy remains regarding the role of stent-graft intervention as a prophylactic procedure in uncomplicated type B dissections^16,17^. Ongoing clinical trials will hopefully shed further light on this subject^18,19^.

The analysis of relative survival is able to provide insights on the impact of AD on longevity of patients affected by this disease. For patients surviving the initial 90 days after an AD event, long-term survival was negatively affected compared with the general matched population, and the effect of AD on long-term relative survival was most prominent in women across all categories. Patients with TAAD undergoing surgical correction had the best chance of achieving a relative survival close to a matched population; still, women had poorer survival than men (10-year relative survival: men 94.9% and women 86.6%). For those undergoing medical management, as well as for TBAD undergoing surgery, the long-term survival was affected more severely, in the range of 85% of that in a matched general population for men at 10 years and 70% for women. Sensitivity analyses demonstrated that some of the poor outcomes could be attributed to age. This reflects women presenting with AD at a later age than men, pushing clinical decisions towards a more conservative approach. Thus, women fare worse when affected by AD, in similarity with many other cardiovascular pathologies^20^. There is also the possibility that women were undertreated with surgical management, or that treatment occurred at a later stage of the pathology. If the same criteria for surgical treatment are applied for women and men (for example diameter threshold for chronic dissection dilatation), this could potentially lead to poorer outcomes due to undertreatment of women. It is also possible that women may be selected for surgical treatment when their anatomy or pathology is more unfavourable than that of the equivalent man, leading to poorer relative long-term survival. Importantly, the evaluation of relative survival in men and women matched for year and age at time of AD presentation, thus removing the impact of management trends and age difference between the sexes on survival.

Data from the present analysis suggest that outcomes after AD have improved over time, with improved relative survival rates for both men and women in the latter interval. Long-term survival after AD is primarily dependent on adequate cardiovascular and hypertension management, as well as adequate imaging follow-up to detect and manage possible late aortic complications, such as aneurysmal degeneration of the chronically dissected aorta. Whilst the present study did not assess changes in medical management of patients with AD in Sweden during the study interval, evaluation of compliance with medical therapy and follow-up may be possible through large national cohorts. This could assist in establishing more grounded evidence in medical management while there is limited agreement on the standards of optimal medical therapy^21^. Recent registry-based research has demonstrated significant benefit for the inclusion of statin therapy in optimal medical management, which highlights the need for further research to shed light on how post-dissection management can be further improved^4^.

As with other large AD epidemiology research that relies on administrative data in the form of diagnosis and procedure coding, reliability of the data collected is of great importance. Evaluation of the Swedish NPR suggests that positive predictive values (PPVs) for diagnostic codes in the NPR are 84% (interquartile range 72–93%), with PPVs for surgical procedures being as high as 97%^22^. Having a specified start date for data inclusion impacts the ability to identify ‘new’ dissections. Accordingly, a washout interval of 5 years was utilized from 1994 where patients with dissections in that interval were excluded.

Specific to the coding used for ADs, there is a significant lack of granularity, making it difficult to classify dissections based on clinical scenario (complicated compared with uncomplicated type B dissection). It is also difficult to delineate the type of dissection in medically managed cases and may have led to inflated event rates with unrecognized inclusion of TAAD within the medically managed cohort. It was impossible to identify incidental diagnosis of chronic ADs that initially had no symptoms and exclude them, so all first diagnoses of AD after the washout interval from 1994 to 1999 were considered the first diagnosis of an acute AD. This may have slightly biased the results. With changes in the NOMESCO coding system in 2021 within Sweden to include more specific details in the surgically treated region, there is the likelihood of an impact on data collection following these changes.

Finally, not all persons who die without admission are warranted an autopsy; this could result in an underestimation of AD-related deaths before admission, as sudden deaths without autopsy may inadvertently be registered as caused by a cardiac event.

## Supplementary Material

znag101_Supplementary_Data

## Data Availability

Data are not publicly shared due to privacy and ethical restrictions, but can be obtained from the corresponding author upon reasonable request.
