# Supplementary material for: Aortic dissection incidence, management, and outcomes: national population-based study
Source: Br J Surg. 2026 Jul 31;113(8):znag101. doi: 10.1093/bjs/znag101 (PMC13505866; doi:10.1093/bjs/znag101)
Supplement: Supplementary file 1 [file znag101_supplementary_data.docx]

Aortic dissection incidence, management and outcomes: a national population-based study

^1^Saricilar, Erin C. *BMed MD MM MS*

^2^Smedberg, Christian, *MD PhD*

^3,4^Hultgren, Rebecka, *MD PhD*

^5^Budtz-Lilly, Jacob, *MD PhD*

^1^Mani, Kevin, *MD PhD FEBVS*

*Affiliation:*

^1^Department of Surgical Sciences

Uppsala University

Uppsala, Sweden

^2^Department of Clinical Science and Education

Södersjukhuset, Karolinska Institet

Stockholm, Sweden

^3^Department of Vascular Surgery

Karolinska University Hospital

Stockholm, Sweden

^4^Vascular Surgery Group

MMK, Karolinska Institutet

Stockholm, Sweden

^5^Department of Cardiothoracic and Vascular Surgery

Aarhus University,

Aarhus, Denmark

**Corresponding author.**

Dr. Erin C. Saricilar

Uppsala University

Sjukhusvägen, Uppsala, Sweden

Email: erin.saricilar@uu.se

**Supplementary Materials - Index**

| **Supplementary Figures and Tables** |  |
| --- | --- |
| Supplementary Figures | *pag. 2* |
| Supplementary Tables | *pag. 9* |

**Supplementary Figures and Tables**

**Supplementary Figures:**

Supplementary Figure S1: Stacked incidence of cases per 100,000 persons per year, delineating male and female cohorts. A summary table of incidences is included underneath the graph.

Supplementary Figure S2: Stacked incidence of cases per 100,000 persons per year, delineating patients less than 65 years old at the time of the dissection and those 65 years old and older. A summary table of incidences is included underneath the graph.

Supplementary Figure S3: a) Kaplan-Meier plot for 10 year survival for medically managed aortic dissections stratified by sex with statistical significance assessed with a log-rank test and showing the number at risk and b) landmark analysis for 10 year survival for medically managed aortic dissections assuming 90 day survival stratified by sex with statistical significance assessed with a log-rank test and showing the number at risk.

Supplementary Figure S4: a) Kaplan-Meier plot for 10 year survival for surgically managed type A aortic dissections stratified by sex with statistical significance assessed with a log-rank test and showing the number at risk and b) landmark analysis for 10 year survival for surgically managed type A aortic dissections assuming 90 day survival stratified by sex with statistical significance assessed with a log-rank test and showing the number at risk.

Supplementary Figure S5: a) Kaplan-Meier plot for 10 year survival for surgically managed type B aortic dissections stratified by sex with statistical significance assessed with a log-rank test and showing the number at risk and b) landmark analysis for 10 year survival for surgically managed type B aortic dissections assuming 90 day survival stratified by sex with statistical significance assessed with a log-rank test and showing the number at risk.

Supplementary Figure S6: Kaplan-Meier plot for males diagnosed with aortic dissection stratified by diagnosis prior to 2009 and from 2009 onwards with statistical significance assessed with a log-rank test for a) 90-day survival and b) 10-year survival assuming 90-days survived.

Supplementary Figure S7: Kaplan-Meier plot for females diagnosed with aortic dissection stratified by diagnosis prior to 2009 and from 2009 onwards with statistical significance assessed with a log-rank test for a) 90-day survival and b) 10-year survival assuming 90-days survived.

**Supplementary Tables:**

Supplementary Table S1: Means and frequencies of all male patient factors including age, region and comorbidities all separated by total dissections, medical management, surgically managed type A aortic dissection and surgically managed type B aortic dissection. Statistics are provided as means (standard deviation) for continuous variables and as frequencies (percentage) for categorical variables. Statistical significance for means were assessed using ANOVA for continuous variables and a Chi-Squared test for categorical variables.

|  | **Medical management** | **Type A surgical management** | **Type B surgical management** | **Total** | **P value** |
| --- | --- | --- | --- | --- | --- |
| **Age**   - < 50 - 50-60 - 60-70 - 70-80 - 80-90 - > 90 | 71.03 (12.46)  267 (5.7%)  545 (11.6%)  1045 (22.2%)  1585 (33.6%)  1118 (23.7%)  152 (3.2%) | 60.66 (11.75)  425 (17.4%)  643 (26.3%)  755 (30.9%)  548 (22.4%)  71 (2.9%)  0 (0.0%) | 63.18 (11.68)  66 (10.8%)  158 (25.9%)  187 (30.7%)  162 (26.6%)  36 (5.9%)  1 (0.2%) | 67.15 (13.11)  758 (9.8%)  1346 (17.3%)  1987 (25.6%)  2295 (29.6%)  1225 (15.8%)  153 (2.0%) | < 0.001  < 0.001 |
| **Region**   - Urban - Metropolitan - Rural | 1325 (28.2%)  1894 (40.3%)  1479 (31.5%) | 786 (32.3%)  950 (39.0%)  701 (28.8%) | 147 (24.1%)  259 (42.5%)  203 (33.3%) | 2258 (29.2%)  3103 (40.1%)  203 (33.3%) | < 0.001 |
| **Comorbidities**   - Cardiac disease - Pulmonary disease - Cerebrovascular disease - Diabetes mellitus - Chronic kidney disease - Cancer - Dementia - Liver disease - Hypertension - Peripheral arterial disease | 2565 (54.4%)  666 (14.1%)  1278 (27.1%)  468 (9.9%)  617 (13.1%)  1047 (22.2%)  238 (5.1%)  228 (4.8%)  2757 (58.5%)  588 (12.5%) | 1186 (48.6%)  199 (8.1%)  553 (22.6%)  118 (4.8%)  324 (13.3%)  287 (11.8%)  111 (4.5%)  104 (4.3%)  1186 (48.6%)  153 (6.3%) | 174 (28.5%)  71 (11.6%)  120 (19.7%)  30 (4.9%)  71 (11.6%)  79 (13.0%)  13 (2.1%)  31 (5.1%)  362 (59.3%)  61 (10.0%) | 3925 (50.6%)  936 (12.1%)  1951 (25.1%)  616 (7.9%)  1012 (13.0%)  1413 (18.2%)  362 (4.7%)  363 (4.7%)  4305 (55.4%)  802 (10.3%) | < 0.001  < 0.001  < 0.001  < 0.001  0.554  < 0.001  0.005  0.482  < 0.001  < 0.001 |

Supplementary Table S2: Means and frequencies of all female patient factors including age, region and comorbidities all separated by total dissections, medical management, surgically managed type A aortic dissection and surgically managed type B aortic dissection. Statistics are provided as means (standard deviation) for continuous variables and as frequencies (percentage) for categorical variables. Statistical significance for means were assessed using ANOVA for continuous variables and a Chi-Squared test for categorical variables.

|  | **Medical management** | **Type A surgical management** | **Type B surgical management** | **Total** | **P value** |
| --- | --- | --- | --- | --- | --- |
| **Age**   - < 50 - 50-60 - 60-70 - 70-80 - 80-90 - > 90 | 73.91 (13.06)  158 (5.3%)  227 (7.6%)  479 (16.1%)  963 (32.4%)  965 (32.5%)  179 (6.0%) | 65.53 (10.89)  105 (8.5%)  208 (16.8%)  420 (34.0%)  430 (34.8%)  74 (6.0%)  0 (0.0%) | 66.26 (13.37)  28 (11.1%)  38 (15.0%)  64 (25.3%)  97 (38.3%)  24 (9.5%)  2 (0.8%) | 71.15 (13.11)  291 (6.5%)  473 (10.6%)  963 (21.6%)  1490 (33.4%)  1063 (23.8%)  181 (4.1%) | < 0.001  < 0.001 |
| **Region**   - Urban - Metropolitan - Rural | 860 (29.0%)  1215 (41.0%)  887 (29.9%) | 365 (29.6%)  537 (43.5%)  333 (27.0%) | 48 (19.0%)  126 (49.8%)  79 (31.2%) | 1273 (28.6%)  1878 (42.2%)  1299 (29.2%) | 0.002 |
| **Comorbidities**   - Cardiac disease - Pulmonary disease - Cerebrovascular disease - Diabetes mellitus - Chronic kidney disease - Cancer - Dementia - Liver disease - Hypertension - Peripheral arterial disease | 1506 (50.7%)  628 (21.1%)  762 (25.6%)  302 (10.2%)  363 (12.2%)  619 (20.8%)  199 (6.7%)  155 (5.2%)  1829 (61.6%)  421 (14.2%) | 625 (50.5%)  163 (13.2%)  295 (23.8%)  52 (4.2%)  127 (10.3%)  171 (13.8%)  60 (4.9%)  55 (4.4%)  675 (54.6%)  81 (6.5%) | 76 (30.0%)  45 (17.8%)  52 (20.6%)  13 (5.1%)  22 (8.7%)  38 (15.0%)  8 (3.2%)  9 (3.6%)  147 (58.1%)  28 (11.1%) | 2207 (49.5%)  836 (18.7%)  1109 (24.9%)  367 (8.2%)  512 (11.5%)  828 (18.6%)  267 (6.0%)  219 (4.9%)  2651 (59.4%)  530 (11.9%) | < 0.001  < 0.001  0.124  < 0.001  0.070  < 0.001  0.011  0.339  < 0.001  < 0.001 |
